# Supplementary material for: A study on the determination of the factors affecting the happiness levels of older individuals during the COVID-19 pandemic in Turkish society
Source: PLoS One. 2025 Jan 23;20(1):e0316000. doi: 10.1371/journal.pone.0316000 (PMC11756808; doi:10.1371/journal.pone.0316000)
Supplement: Attachment — Submitted filename: PONE-D-2340834 REVIWER REPORT revised on 20-5-2024.docx [file pone.0316000.s001.docx]

**Dear Authors,**

Thank you for your efforts in revising the manuscript titled "A Study on the Determination of the Factors Affecting the Happiness Levels of Older Individuals During the COVID-19 Pandemic in Turkish Society."

**Overall Analysis:**

The manuscript analyzes data collected by TURKSTAT in 2020, focusing on factors influencing the happiness levels of elderly individuals in Türkiye during the COVID-19 pandemic. This analysis offers valuable insight into the socio-psychological trends among Turkish seniors during this challenging period. While the studied factors have been explored previously in both Turkish and non-Turkish populations, your focus on the pandemic sheds light on the unique struggles, concerns, and challenges faced by older adults during this stressful time.

**Minor Suggestions:**

1. **Data Subgroup Analysis:** While I am not a statistical expert, I recommend considering dividing the data of 1863 individuals into age groups (e.g., 60-69, 70-79, 80-89) for a more precise analysis of the studied factors. This would allow for a nuanced understanding of happiness across different age segments within the elderly population.
2. **Happiness Factors by Region:** In the discussion of happiness factors, consider categorizing references geographically (e.g., continents, developed vs. developing countries) to highlight potential similarities or differences based on socio-economic characteristics. This would provide a more structured approach to the existing analysis.
3. **Reference Refinement:** In lines 129-132, you mention research conducted in the Americas and South Africa. While both regions fall under the Americas classification, there might be significant cultural and socio-economic differences. For a more focused comparison, consider referencing research specific to South Africa or separately addressing research from the Americas and South Africa.

Additionally, I suggest Hou et al. (2023) as a potential reference – a valuable addition to support the discussion on religious belief and happiness.

Hou B, Wu Y and Huang Y. How Does Religious Belief Affect Happiness of Older Adults: The Participation Effect and Support Effect. Religions.2023; 14: 243. <https://doi.org/10.3390/rel14020243>

4. **Explaining Results:** In the discussion section, consider providing explanations for some of the findings. For example, line 370-371 suggests individuals with less education might be happier than those with a university degree. Including a possible explanation for this result would strengthen your analysis.

The same approach could be applied to other unexpected findings to enrich the discussion.

**Thank you again for your work. These suggestions aim to further enhance the clarity and impact of your manuscript.**

**Sincerely,**

Assist. Prof. Luma H.A. AL Obaidy
