# Supplementary material for: A study on the determination of the factors affecting the happiness levels of older individuals during the COVID-19 pandemic in Turkish society
Source: PLoS One. 2025 Jan 23;20(1):e0316000. doi: 10.1371/journal.pone.0316000 (PMC11756808; doi:10.1371/journal.pone.0316000)
Supplement: Attachment — Submitted filename: Response to reviewers-12.07.2024.docx [file pone.0316000.s002.docx]

**Dear Editor and Reviewers,**

Thank you very much for your comments concerning our manuscript titled “A Study on the Determination of the Factors Affecting the Happiness Levels of Older Individuals During the COVID-19 Pandemic in Turkish Society”. These comments have been very helpful in reviewing and improving our manuscript. We have carefully revising these instructive comments and made corrections that we hope will be approved. The revised parts are highlighted in red in the main paper.

The corrections to the manuscript and responses to the reviewer’s comments are as following:

Regards,

Authors

**Review Comments to the Author**

Please use the space provided to explain your answers to the questions above. You may also include additional comments for the author, including concerns about dual publication, research ethics, or publication ethics. (Please upload your review as an attachment if it exceeds 20,000 characters).

**Reviewer #1:**

I am pleased that you have invited me to review a manuscript titled “A Study on the Determination of the Factors Affecting the Happiness Levels of Older Individuals During the COVID-19 Pandemic in Turkish Society”. The manuscript is easy to read and understand. Also, the manuscript is nicely presented, following a thorough reading and comprehension of the paper, I forwarded the following comments.

**Reviewer point #1:** There are numerous typographical and grammatical issues that should be handled.

**Author response #1:** Thank you for the comment. Language and grammatical errors were corrected during the “proofreading” made by the language editor.

**Reviewer point #2:** In the abstract section “1,863 individuals aged 60 and participated in this survey”. Write as 60 and above.

**Author response #2:** Thank you for the comment. Taking this criticism into account, we have revised the statement.

**Reviewer point #3:** From lines 257-260 the manuscript mentions about chi-square test and it says, that the test not only determines the significance of observed differences but also provides information about the specific categories where differences occur. Is that true? The chi-square test didn’t provide any information about in which category the differences occur. It should be corrected.

**Author response #3:** Thank you for the comment. Taking this criticism into account, we have revised the sentence.

**Reviewer point #4:** Regarding Education Level, the categories are not exhaustive. What about those who have a Diploma, and TVET colleges’ certificate, MSc and above? What does that mean no degree?

**Author response #4:** Thank you for the comment. Taking this criticism into account, we separated the categories on the education level variable and re-estimated the model. We reinterpreted the results according to the new model. Changed to “did not complete a school”.

**Reviewer point #5:** Categories of some variables are ambiguous. If we see the variable “Source of Happiness as a Person”: (Himself/herself/spouse, Children/mother/father, Friends/ nephews/ grandchildren/ other, All family members), and “Source of Happiness”: (Success, Job/money/other, Health, Love), you have used the word other. This leads to confusion. Because if we use this term (other), all other categories are also included which leads miss conception. Should take measures.

**Author response #5:** Thank you for the comment. Taking this criticism into account, we have revised the statement.

**Reviewer point #6:** Even if in line 305 you expressed the absence of a multicollinearity problem between the variables, variables like Hope for the future and Hope full scale are nearly similar and redundant variables. In addition frequency for neither hopeful nor not hopeful is missed. So better if you use either variable only.

**Author response #6:** Thank you for the comment. Taking this criticism into account, we removed the Hope for the future variable and re-estimated the model. We reinterpreted the results according to the new model.

**Reviewer point #7:** In lines 336-337, it says that “Individuals with very satisfied/satisfied health satisfaction are 10.8% less likely to be “moderately happy” in the reference category”. What does that mean? Re-write it. Similarly for a statement on lines 339-340, “The probability of being “very happy/happy is 19.6% and 22.2% lower than the hopeful reference category”.

**Author response #7:** Thank you for the comment. Taking this criticism into account, we have revised the statement.

**Reviewer point #8:** The major issue I saw in this study is the lack of justification. For example, the results of the study state that “men are less likely to be very happy/happy than women. …. Contrary to this study, some studies concluded that men are happier than women [34, 75]”. This needs justification as to why men are less likely to be “very happy/happy” than women and why the results of this study contradict with other studies done in different places at different times. Similarly for all the rest variables.

**Author response #8:** Thank you for the comment. Taking this criticism into account, we have revised the discussion. The discussion has been rewritten.

**Reviewer #2:**

Referee report for “A Study on the Determination of the Factors Affecting the Happiness Levels of Older Individuals During the COVID-19 Pandemic in Turkish Society”

Summary

The study aimed to identify factors influencing the happiness levels of older individuals in Turkey during the COVID-19 pandemic. It utilized data from the 2020 Life Satisfaction Survey conducted by TURKSTAT, involving 1,863 participants aged 60 and above. The relationship between happiness levels and various factors was analyzed using the chi-square independence test and generalized ordered logistic regression analysis. Results showed that individuals aged 60-64 were 10.1% less likely to be happy compared to those aged 65 and older, while men were 4.3% less likely to be happy than women. Moreover, individuals with lower education levels had higher probabilities of happiness, and those hopeful about the future were less likely to be happy. The study identified gender, age, education, source of happiness, health satisfaction, hope scale, and homeownership as factors impacting happiness levels in older individuals, suggesting societal support as an indicator of happiness.

Comments to author

Major issues:

**Reviewer point #1:** The introduction provides a general overview of happiness and its importance but lacks a clear statement of the research objectives. The abstract and introduction should clearly outline the aim of the study, which seems to be identifying factors influencing the happiness levels of older individuals during the COVID-19 pandemic in Turkey. However, this should be explicitly stated.

**Author response #1:** Thank you for the comment. Taking this criticism into account, we have added the necessary explanations in introduction.

**Reviewer point #2:** While the study mentions various factors affecting happiness, it lacks a theoretical framework that would provide a structured understanding of how these factors interact and influence happiness levels among older individuals during the pandemic.

**Author response #2:** Thank you for the comment. Taking this criticism into account, we have revised the introduction. We have added the necessary explanations in introduction.

**Reviewer point #3:** The methods section lacks detail on certain crucial aspects, such as the selection criteria for the sample and the specific measures used to assess happiness levels. Additionally, the description of the statistical analysis could be more detailed to provide clarity on the modeling approach employed.

**Author response #3:** Thank you for the comment. Taking this criticism into account, we have revised the Data and Statistical Analysis sections. We have added the Figure 1 for for the sample.

**Reviewer point #4:** The study relies on self-reported data from the Life Satisfaction Survey, which may introduce bias due to participants' subjective interpretations of their happiness levels.

**Author response #4:** Thank you for the comment. Taking this criticism into account, we have revised the Limitations of the study.

**Reviewer point #5:** The study's cross-sectional design limits its ability to establish causal relationships between happiness and socio-economic factors. It acknowledges this limitation but does not propose avenues for further longitudinal research.

**Author response #5:** Thank you for the comment. Taking this criticism into account, we have revised the Limitations of the study.

**Reviewer point #6:** The study lacks information on participants' COVID-19 status, which could be crucial in understanding the pandemic's direct impact on happiness levels.

**Author response #6:** Thank you for the comment. The survey did not ask participants about their COVID-19 status. This limitation was stated in the study. Taking this criticism into account, we have revised the Limitations of the study.

**Reviewer point #7:** While the study provides insights into the factors affecting the happiness of older individuals in Turkey during the COVID-19 pandemic, its findings may not be directly applicable to other cultural or demographic contexts.

**Author response #7:** Thank you for the comment. Taking this criticism into account, we have revised the Limitations of the study.

**Reviewer point #8:** While the study employs statistical methods like chi-square tests and logistic regression, it lacks detailed discussion on the assumptions underlying these techniques and potential limitations.

**Author response #8:** Thank you for the comment. Taking this criticism into account, we have revised the Data and Statistical Analysis sections.

**Reviewer point #9:** While the study presents regression coefficients and odds ratios, there's a lack of detailed interpretation of these results. Providing more context and discussing the practical implications of the findings would enrich the discussion section.

**Author response #9:** Thank you for the comment. We interpreted the marginal effects. Taking this criticism into account, we have revised the discussion. The discussion has been rewritten.

Minor issues:

**Reviewer point #10:** There are minor typographical errors, such as missing punctuation marks (e.g., comma after "In addition" in line 202) and inconsistencies in capitalization (e.g., "Türkiye" vs. "TURKSTAT"). Proofreading for such errors would enhance the readability of the manuscript.

**Author response #10:** Thank you for the comment. Language and grammatical errors were corrected during the “proofreading” made by the language editor.

The United Nations has decided to use “Türkiye” instead of “Turkey” internationally as of June 2022. Due to this decision, the word “Türkiye” was used in the manuscript.

(https://turkiye.un.org/en/184798-turkeys-name-changed-t%C3%BCrkiye)

**Reviewer point #11:** Certain sections could be clearer, especially in explaining the rationale behind the choice of variables and the interpretation of results.

**Author response #11:** Thank you for the comment. Taking this criticism into account, we have revised the results.

**Reviewer point #12:** The discussion section briefly touches on each variable but could benefit from a deeper exploration of the literature and theoretical frameworks supporting the study's findings.

**Author response #12:** Thank you for the comment. Taking this criticism into account, we have revised the discussion. The discussion has been rewritten.

**Reviewer point #13:** There are occasional grammatical errors and awkward phrasings that could be smoothed out for clarity and readability.

**Author response #13:** Thank you for the comment. Language and grammatical errors were corrected during the “proofreading” made by the language editor.

**Reviewer point #14:** The study does not explicitly mention any limitations or potential biases in the methodology or data analysis. Acknowledging these limitations would provide transparency and help readers interpret the findings more accurately.

**Author response #14:** Thank you for the comment. Taking this criticism into account, we have revised the Data and Statistical Analysis sections.

**Reviewer point #15:** The study briefly mentions the need for further research but could provide more specific suggestions for future studies, such as exploring longitudinal data or conducting qualitative research to complement quantitative findings.

**Author response #15:** Thank you for the comment. Taking this criticism into account, we have added the necessary explanations in conslucios.

**Reviewer #3:**

Dear Authors,

Thank you for your efforts in revising the manuscript titled "A Study on the Determination of the Factors Affecting the Happiness Levels of Older Individuals During the COVID-19 Pandemic in Turkish Society."

Overall Analysis:

The manuscript analyzes data collected by TURKSTAT in 2020, focusing on factors influencing the happiness levels of elderly individuals in Türkiye during the COVID-19 pandemic. This analysis offers valuable insight into the socio-psychological trends among Turkish seniors during this challenging period. While the studied factors have been explored previously in both Turkish and non-Turkish populations, your focus on the pandemic sheds light on the unique struggles, concerns, and challenges faced by older adults during this stressful time.

Minor Suggestions:

**Reviewer point #1:** Data Subgroup Analysis: While I am not a statistical expert, I recommend considering dividing the data of 1863 individuals into age groups (e.g., 60-69, 70-79, 80-89) for a more precise analysis of the studied factors. This would allow for a nuanced understanding of happiness across different age segments within the elderly population.

**Author response #1:** Thank you for the comment. Taking this criticism into account, we separated the categories on the age variable and re-estimated the model. We reinterpreted the results according to the new model.

**Reviewer point #2:** Happiness Factors by Region: In the discussion of happiness factors, consider categorizing references geographically (e.g., continents, developed vs. developing countries) to highlight potential similarities or differences based on socio-economic characteristics. This would provide a more structured approach to the existing analysis.

**Author response #2:** Thank you for the comment. Taking this criticism into account, we have revised the Literature.

**Reviewer point #3:** Reference Refinement: In lines 129-132, you mention research conducted in the Americas and South Africa. While both regions fall under the Americas classification, there might be significant cultural and socio-economic differences. For a more focused comparison, consider referencing research specific to South Africa or separately addressing research from the Americas and South Africa.

Additionally, I suggest Hou et al. (2023) as a potential reference – a valuable addition to support the discussion on religious belief and happiness.

Hou B, Wu Y and Huang Y. How Does Religious Belief Affect Happiness of Older Adults: The Participation Effect and Support Effect. Religions.2023; 14: 243. https://doi.org/10.3390/rel14020243

**Author response #3:** Thank you for the comment. Taking this criticism into account, we have revised the Literature and Discussion. We examined the relevant references in detail.

**Reviewer point #4:** Explaining Results: In the discussion section, consider providing explanations for some of the findings. For example, line 370-371 suggests individuals with less education might be happier than those with a university degree. Including a possible explanation for this result would strengthen your analysis.

The same approach could be applied to other unexpected findings to enrich the discussion.

Thank you again for your work. These suggestions aim to further enhance the clarity and impact of your manuscript.

Sincerely,

**Author response #4:** Thank you for the comment. Taking this criticism into account, we have revised the Results and Discussion.
