# Supplementary material for: A study on the determination of the factors affecting the happiness levels of older individuals during the COVID-19 pandemic in Turkish society
Source: PLoS One. 2025 Jan 23;20(1):e0316000. doi: 10.1371/journal.pone.0316000 (PMC11756808; doi:10.1371/journal.pone.0316000)
Supplement: Attachment — Submitted filename: Response to reviewers-15.10.2024.docx [file pone.0316000.s003.docx]

**Dear Editor and Reviewers,**

Thank you very much for your comments concerning our manuscript titled “A Study on the Determination of the Factors Affecting the Happiness Levels of Older Individuals During the COVID-19 Pandemic in Turkish Society”. These comments have been very helpful in reviewing and improving our manuscript. We have carefully revising these instructive comments and made corrections that we hope will be approved. The revised parts are highlighted in red in the main paper.

The corrections to the manuscript and responses to the reviewer’s comments are as following:

Regards,

Authors

**Reviewer #3:**

To the authors,

**Reviewer point #1:** Thank you for completing the required amendments in the review and I would like to point out that the conclusions should be limited to the factors studied in the study.

Best wishes

**Author response #1:** Thank you for the comment. Taking this criticism into account, we have added “*The conclusions were limited to the factors studied in the study*” to the Limitations of the study section.

**Reviewer #4:**

**Reviewer point #1:** Thank you for the opportunity to review your manuscript. The study examined the factors influencing happiness levels among different demographic groups in Turkey during the COVID-19 pandemic. While I appreciate the efforts put into conducting this research, I have observed significant overlaps in the overarching themes, methodologies, and some aspects of the analysis with another recently published work, by some of the same authors (https://doi.org/10.5455/PBS.20230512053548).

Such similarities raise concerns about the originality and incremental contribution of this manuscript. It is crucial for the advancement of scholarly work that each publication offers a distinct and significant contribution to the existing body of knowledge. In light of this, I would recommend clarifying how this manuscript distinctly advances our understanding compared to previously published works, especially those conducted using the same dataset and similar methodology.

**Author response #1:** Thank you for the comment. In this study, data from elderly individuals over 60 years of age, one of the disadvantaged groups during the COVID-19 pandemic period, were used. Taking this criticism into account, we have revised the Introduction. We have added the following statements to the introduction.

“*The older adults are at-risk groups during the COVID-19 pandemic. Identifying the factors affecting the happiness levels of older individuals during the COVID-19 period is of great importance for developing more targeted strategies to minimize the adverse effects of the pandemic on the elderly and protect their quality of life. It is important to improve the quality of life of older individuals and provide a better ageing process. COVID-19 has been a disease that carries serious health risks for older individuals. The uncertainties of COVID-19 and health concerns have led to an increase in mental health problems such as anxiety, depression and stress in older adults. To cope with these problems, it is critical to identify the factors that affect happiness and adapt mental health services accordingly. Therefore, identifying the factors that affect the happiness levels of older individuals to protect their physical and psychological health may help reduce the disease's adverse effects.*

*The findings indicate that several factors affect the happiness levels of those aged 60 and older. According to the research, factors such as gender, age, education level, income level, and being hopeful affect the happiness of older individuals. It is essential to know these factors to design more effective social policy and health services according to the needs of the elderly population. Thus, better health services, social assistance and education programs can be developed for older adults. To better understand the relationship between happiness-related factors, a larger study sample is needed among older individuals whose lives were changed by strict isolation methods during the COVID-19 period in Türkiye. For this reason, the purpose of this study is to review the current policies throughout the country, to re-evaluate the needs that arise in the face of extraordinary altering circumstances such as the COVID-19 pandemic, and to fill this gap in the literature.*

*Understanding the factors affecting the happiness levels of older individuals during the COVID-19 period can increase the effectiveness of the measures and support mechanisms to be taken to help them overcome this difficult period with less damage and live a healthier and happier old age.*”

**Reviewer point #2:** In addition, I have a major concern about the reproducibility of the current findings. Although the raw data may be subject to third-party restrictions in reproduction, providing the statistical scripts and models used in the research would enhance transparency and verifiability. This is crucial for allowing future researchers to validate and reproduce your findings and facilitates comparative studies in cross-cultural or similar contexts. I recommend including the scripts for data preprocessing and analysis in the supplementary materials.

Thank you for addressing these concerns.

**Author response #2:** Thank you for the comment. Taking this criticism into account, we have revised the paper. We have added the following statements.

“*The data were obtained through the joint teamwork of both the Turkish Statistical Institute (TSI) and the European Union Statistical Office (SOEU). We obtained this data from TSI in return for a contract without needing an ethics committee document and used it in our study.*

*TSI is an institution that compiles, evaluates, and presents statistical information to decision-makers to prepare development plans and programs, make economic decisions, and address all other issues needed. TSI carries out internationally comparable statistical production activities according to the standards of organizations such as the European Union Statistical Office, the United Nations, OECD, ILO, etc. TSI collects data within the scope of the Official Statistics Program. The Official Statistics Program is prepared for five-year periods based on the Turkish Statistics Law No. 5429 to determine the basic principles and standards regarding the production and publication of official statistics and to ensure the production of up-to-date, reliable, timely, transparent and impartial data in areas of need at national and international levels [128]. TSI also conducts the Türkiye Health Survey within the scope of the Official Statistics Program put into effect by law. Since the Türkiye Health Survey is conducted within the scope of legal responsibility by the state, ethical approval is not required [129].*

*For this study, secondary data were employed. Official approval was received from the Turkish Statistical Institute to use the microdata set from the Life Satisfaction Survey. The Turkish Statistical Institute also received a “Letter of Undertaking” authorizing it to use the study’s data.*

*The letter of undertaking for the use of micro data without restrictions in dissemination:*

*Article 1- This letter of undertaking determines the rules, principles and obligations of the use of micro data, which are safe to disclose apart from the Presidency.*

*Article 2-This letter of undertaking regulates the use of micro data sets of Life Satisfaction Survey, 2020, within the framework of the Directive on Access and Use of Micro Data in line with the purpose specified in Article 1.*

*Article 3- The following provisions apply for the use of micro data:*

*a)	Findings obtained by the researcher as a result of incorrect calculation only bind the researcher.*

*b)	The researcher refers to the micro data of the Institution that he uses while disclosing the results obtained from the study.*

*c)	The researcher is obliged to send a copy of the published report, article, publication etc. to the Institution Library within three months at the latest. Subsequent micro data usage requests of the researcher who is found not to fulfill this obligation are not covered.*

*d)	The researcher cannot reproduce, give to third parties, sell or transfer the micro data set he obtained.*

*Article 4-The researcher, by taking into account the principles of confidentiality defined in 13. and 14. articles of Turkish Statistical Institution numbered 5429 and Regulation on Procedures and Principles Regarding Data Confidentiality and Confidential Data Security in Official Statistics, is deemed to guarantee hereby that he shall not disclose the information, table, etc. violating this principle and shall only use micro data for statistical purposes.*”
